# Highly sensitive magnetite nano clusters for MR cell imaging

**DOI:** 10.1186/1556-276X-7-204

**Published:** 2012-03-31

**Authors:** Mingli Li, Hongchen Gu, Chunfu Zhang

**Affiliations:** 1 School of Biomedical Engineering & Med-X Research Institute, Shanghai Jiao Tong University, Shanghai 200030, China

**Keywords:** Magnetic resonance imaging, magnetic nano cluster, cell imaging, polyol method

## Abstract

High sensitivity and suitable sizes are essential for magnetic iron oxide contrast agents for cell imaging. In this study, we have fabricated highly MR sensitive magnetite nanoclusters (MNCs) with tunable sizes. These clusters demonstrate high MR sensitivity. Especially, water suspensions of the MNCs with average size of 63 nm have transverse relaxivity as high as 630 s^-1^mM^-1^, which is among the most sensitive iron oxide contrast agents ever reported. Importantly, such MNCs have no adverse effects on cells (RAW 264.7). When used for cell imaging, they demonstrate much higher efficiency and sensitivity than those of SHU555A (Resovist), a commercially available contrast agent, both in vitro and in vivo, with detection limits of 3,000 and 10,000 labeled cells, respectively. The studied MNCs are sensitive for cell imaging and promising for MR cell tracking in clinics.

## Background

Cell imaging is very important for cell-based therapies and has attracted increasing attention in recent years. Due to its high spatial resolution in three dimensions and good soft-tissue contrast, MR imaging is highly desirable for this purpose and has been demonstrated to be a robust tool for imaging and tracking the migration of stem cells in various diseases [1,2]. For MR cell imaging, cells need to be labeled with magnetic contrast agent to distinguish them from the surrounding tissues by MRI. Currently, the frequently used MR contrast agents are gadolinium-based "positive" contrast agents and superparamagnetic iron oxide nanoparticle (SPION)-based "negative" contrast agents [3,4]. Since gadolinium-based MR contrast agents have a high detectability threshold, the use of SPIONs now provides a more promising alternative to label and detect the target cells [2].

However, for tracking small amount of cells, MR sensitivity of the commercially available SPION agents are still relatively low. In this context, to enhance the MRI detection sensitivity, antibodies, HIV-Tat peptides or transfect agents were often conjugated to or combined with the particles to facilitate nonphagocytic ingestion of them [5]. Because these approaches require the delicate composition of the contrast agents and the "cell-uptake enhancers," novel MR contrast agents that facilitate stem-cell labeling have developed rapidly in recent years [6,7].

In addition to improve the labeling efficiency of the cells, another way to enhance the detection sensitivity is to improve the sensitivity of the contrast agents. In this regard, SPION clusters or SPION-imbedded macro-sized polymer spheres have been explored for cell imaging [8-11]. Especially, for SPION macro spheres, the transverse relaxivities are higher than the commonly used dextran-coated SPION agents by nearly 50% [12] and when used for cell labeling, because of the high iron content per macro shpere, the average intracellular iron of about 100 pg per cell can be achieved [13] Integrating the high sensitivity and iron content into one entity, the macro-sized spheres have been demonstrated high efficiency for cell labeling and tracking [12,14]. Although short-term cytotoxicity of the spheres was not found, due to its large size and direct exposure of the cell with benzene compound coating material, there is considerable concern about the long-term safety of the contrast agents. More over, recent study indicated that high intracellular iron concentration would diminish cell proliferation, disrupt microtubule network and alter the focal adhesion kinase signaling, which would finally induce cell apoptosis [15]. Therefore, developing high sensitive SPION contrast agent with relative lower iron content per particle is highly desired for cell imaging and tracking.

In current study, we have fabricated compact MNCs with different sizes and found that water suspensions of MNCs with average size of 63 nm have the T_2 _relaxivity as high as 630 s^-1^mM^-1^. The MNCs are much more robust for cell imaging than carboxydextran-coated SPION (SHU555A) both in vitro and in vivo.

## Methods

### Synthesis and characterization of magnetite nanoclusters (MNCs)

#### Synthesis of 34 nm, 63 nm, 106 nm and 166 nm MNCs

MNCs were synthesized according to our previous method [16,17]. In brief, FeCl_3 _6H_2_O (3 mmol) and poly (acrylic acid) (PAA, MW 5000, 4 mmol) were dissolved in ethylene glycol (30 mL) to form a uniform solution under ultrasonic and vigorous stirring. After adding de-ionized water (4000, 2000, 250 or 100 μL) and urea (0.3 mol), the solution was ultrasonicated for several minutes and then sealed in a Teflon lined stainless-steel autoclave (50 mL). The autoclave was heated at 200°C for 12 h, and then allowed to cool at ambient temperature. The black products were separated magnetically and were washed several times with ethanol and de-ionized water to eliminate organic and inorganic impurities and dried in vacuum at 60°C for 10 h.

#### Characterizations

The morphology and size of the clusters were observed and analyzed by transmission electron microscopy (TEM, JEOL 2100F, Japan). For this purpose, MNCs suspension was directly deposited onto a carbon-coated copper grid and air-dried at room temperature. The particle sizes and size distributions were calculated using an image analysis program by measuring the diameter of at least 300 particles.

Fourier transform infrared (FT-IR) spectra of the solid samples were recorded on a PerkinElmer spectrum 100 (Perkin Elmer, Massachusetts). All samples were ground and mixed with KBr and then pressed to form pellets. Spectra were recorded in the wave number interval between 4000 and 500 nm^-1^. The background spectrum was subtracted from the sample spectrum. Each spectrum was acquired three times, and an average of the three measurements was taken and analyzed.

To determine the crystal structure and component grain size, powder X-ray diffraction (XRD) patterns were obtained using Cu Ka radiation (λ = 1.540 Å) with a Dmax-r C X-ray diffractometer (Rigaku, Tokyo, Japan) operated at 40 kV and 100 mA. The scanning ranges from 10° to 80° with a speed of 15°/minute. For calculating the size of component grains, the scanning range was from 32° to 46° with a speed of 1°/minute.

Thermal gravimetric analysis were performed by TG209 F1 NETZSCH (NETZSCH, Germany) from 30°C to 1000°C with 10°C per minute under the protection of nitrogen to determine the solid content of the clusters.

The magnetizations of the products were obtained by a vibration sample magnetometer (VSM, Lakeshore 7300) at 1.41 T. The magnetization (*M*, emu/g) of the samples was measured as a function of the magnetic field (*H*, Oe) at 300 K.

The hydrodynamic diameters and zeta potentials of the particles (at pH 7.0) were measured using a Malvern NanoZS spectrometer (NanoZS, Malvern, UK).

Nuclear MR relaxometry of the MNCs were measured at 37°C by an NMR spectrometer (Minispec, mq60, Brucker, Germany) with ac magnetic field frequency of 60 MHz (1.41 T). Carr-Purcell-Meiboom-Gill (CPMG) sequence was adopted and the echo time was set as 0.5 ms. The details of measurement were according to our previous report [18].

### In vitro study

#### Cell culture

RAW 264.7 cell, a macrophage cell line, was purchased from Shanghai Institute for Biological Sciences, Chinese Academy of Sciences (Shanghai, China) and maintained with a DMEM culture medium supplemented with 10% FBS, 100 IU mL^-1 ^penicillin and 100 μg mL^-1 ^streptomycin under 5% CO_2 _atmosphere at 37°C.

#### Cell labeling

For cell labeling, MNCs with average size of 63 nm was used, with SHU555A (Resovist, Schering AG, Germany) as a reference [4]. To determine the concentration-dependent uptake by the macrophage cell line, the cells were incubated with the MNCs or SHU555A at different iron concentrations in culture media (5 μg/mL, 10 μg/mL, 50 μg/mL, 100 μg/mL and 300 μg/mL) at 37°C for 60 minutes. To determine the time-dependent uptake, the cells were incubated with 10 μg/mL (in iron) MNCs or SHU555A for different periods of time (0.5, 1.5, 2, 3, 4, 5 h). After incubation, the cells were washed three times with PBS, trypsinized, and counted in a Neubauer counting chamber for the following experiments.

#### ICP-OES quantification of intracellular iron

To determine the intracellular iron, inductively coupled plasma atom emission spectrometer ((ICP-OES, ICAP-6300, Thermo Fisher, USA) measurements of the labeled cells were performed for both cases. Before measurement, the cells were digested with aqua regia, and diluted to 10 mL by adding de-ionized water. Triplet experiments was performed and the average value of intracellular iron content was taken, and expressed as mean ± SD in picograms of Fe per cell.

#### MR imaging of the labeled cells

For MR imaging, the labeled cells (1 × 10^5^) were suspended in gelatin (2%, 500 μL) in plastic vials homogenously and placed in a water tank. MRI was performed with a 3 T MRI scanner (TrioTim, Siemens, Germany) using a clinical head coil with T_2_-weighted spin echo sequence (TR = 2000 ms, TE = 37 ms, FOV = 220 × 220 mm, slice thickness = 2 mm) and T_2_*-weighted gradient echo sequence (TR = 400 ms, TE = 4.18 ms, FOV 62 × 199 mm, slice thickness = 2 mm). T_2 _and T_2_* relaxation times were determined with a multi slice multi echo MAP MSME spin echo sequence (TR = 1670 ms, TE range = 15~180 ms, 12 echoes, FOV = 62 × 200 mm, slice thickness = 2 mm) and gradient echo sequence (TR = 400 ms, TE range = 4.18~41.44 ms, 10 echoes, FOV = 62 × 200 mm, slice thickness = 2 mm), respectively. The relaxation times were calculated by a linear fit of the logarithmic region-of-interest signal amplitudes versus TE.

To determine the detection threshold, different amount of cells (1 × 10^3^, 3 × 10^3^, 5 × 10^3^, 1 × 10^4^, 5 × 10^4^) treated with 100 μg/mL particles for 1 h were suspended in 300 μL gelatin (2%). T_2_-weighted MR imaging of the cells were performed with the parameters same as the above mentioned.

#### Prussian blue staining

Cells were seeded on cover glass slices and incubated with the MNCs or SHU555A at different iron concentrations (5, 10, 50, 100, 300 μg/mL) for 60 minutes or at 10 μg/mL iron concentration for different periods of times (0.5, 1.5, 2, 3, 4, 5 h). After incubation, cells were washed three times with PBS. For Prussian blue staining, cells were fixed with paraformaldehyde (4%) for 10 min. The fixed cells were stained with 10 wt% Prussian blue (K_4_FeCN)_6 _3H_2_O) for 5 minutes and the mixture of 10 wt% Prussian blue and 20% HCl (1:1) for 30 min successively. After washing with water, the cells were counterstained with nuclear fast red for 5 min. The stained cells were dehydrated with gradient ethanol (70%, 95% and 100%), cleared with xylene and adhered onto glass slides for microscopic study.

#### Viability of labeled cells

The viability of cell was evaluated with MTT method [19]. For this purpose, the cells (1 × 10^4^) were seeded on each well of 96 multi-well plate and incubated with the MNCs at the iron concentrations of 10 μg/mL and 100 μg/mL for different periods of time (1, 5 and 24 h). After incubation, the culture medium was removed and the cells were washed with PBS three times. Subsequently, fresh cell culture medium (90 μL) was added into each well, followed by adding 10 μL MTT solution (5 mg/mL). Then, the cells were incubated for 4 h. After dissolving the formanzan dye produced by live cells with DMSO (100 μL), the absorbance at 570 nm was recorded using a Wallace 1420 multilabel counter VICTOR3 (PerkinElmer, Baltimore, MD). Cell viability was expressed as percentage of the absorbance of cells incubated with probes to that of cells maintained in normal culture medium.

### In vivo study

The animal experiments were approved by the animal protection and care committee of Shanghai Jiao Tong University. To test the potential of the MNCs for cell imaging in vivo and MR detection sensitivity of the labeled cells, cells were treated with the MNCs or SHU555A at the iron concentration of 100 μg/mL for 60 min. The labeled cells (1 × 10^4^, 1 × 10^5^, 1 × 10^6^) were dispersed into Matrigel (400 μL) and imbedded subcutaneously into the flanks of mice (CD-1). MR imaging at coronal position was performed after implantation using a 3 T MRI scanner (TrioTim, Siemens) with T_2_-weighted multi slice multi echo sequence (TR = 3500 ms, TE = 20 ~ 160 ms, 8 echo, FOV 45 × 45 mm, slice thickness = 2 mm).

### Statistical evaluation

Statistical analysis of ICP-OES data of cells treated with the MSCs or SHU555A were conducted using a Student's *t *test. A *p *value of < 0.05 was considered to indicate significant differences between groups.

## Results and discussion

In current study, compact magnetite nanoclusters (MNCs) with various sizes were synthesized by polyol method. MNCs with average size of 63 nm is the most MR sensitive, with the transverse relaxivity of 630 s^-1 ^mM^-1^. When used for cell imaging, the MNCs also demonstrate superior sensitivity than the frequently used SPION contrast agent (SHU555A) both in vitro and in vivo. Our study indicates that the studied MNCs with size of 63 nm are highly sensitive for cell imaging.

### Synthesis and characterization of magnetite nanoclusters (MNCs)

**Figure 1:**
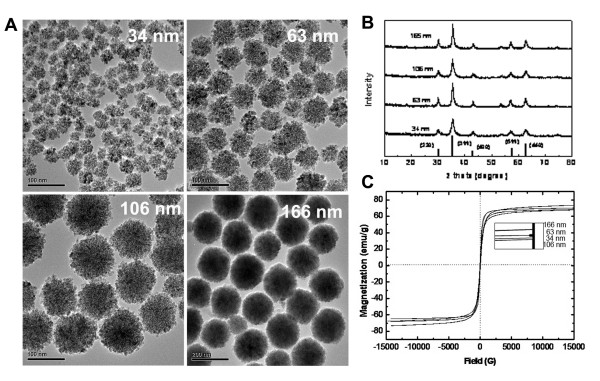
**Characterizations of magnetic nanoclusters (MNCs)**. A: TEM images of MNCs of different sizes. B: XRD pattern of MNCs. C: Magnetic hysteresis loops of MNCs at 300 K. Inset: Magnification of hysteresis curves for distinguishing the differences of saturation magnetizations of different MNCs.

Magnetite nanoclusters were synthesized by polyol process, in which polyacrylic acid (PAA, MW 5000) was used as the stabilizer to improve their dispersity in water [16,20]. By varying the amount of water added in the reaction, clusters with different sizes (34, 63, 106, 166 nm) were obtained. As the size increased, the clusters became more uniform (Figure 1A). Close inspection of the TEM images confirms that these clusters are composed of small primary grains. The crystal structure of the primary grains was identified by using X-ray diffraction (XRD) (Figure 1B). The peaks were labeled with the indexed Bragg reflections of the magnetite structure and the particles were found to be highly crystalline. Using the Debye-Scherrer formula, the average sizes of the crystallite are determined to be 11, 12, 9, 14 nm for 34, 63, 106 and 166 nm clusters, respectively. The magnetization of the clusters was measured by using vibration sample magnetometer (VSM) as a function of applied magnetic field (Figure 1C). After normalized to the mass of solid content, the saturation magnetizations for 34, 63, 106, 166 nm clusters were 68, 69, 67, 74 emu/g, respectively. The residual magnetism and coercivity of clusters are very low. These observations are in line with the above calculations for the sizes of component particle that are all less than 15 nm.

**Figure 2:**
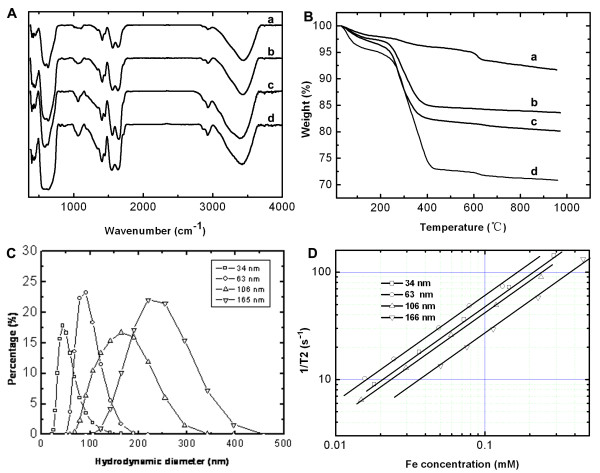
**A and B: FT-IR spectra and TGA curves of 34 nm (a), 63 nm (b), 106 nm (c) and 166 nm (d) MNCs**. C: Volume-averaged hydrodynamic size of MNCs. The average sizes are 53, 95, 160, 236 nm, respectively for 34, 63, 108, 166 nm clusters. D: T_2 _relaxation rates (1/T_2_) plotted against the iron concentrations for the MNCs. T_2 _relaxavity of the clusters are 490 (34 nm), 630 (63 nm), 420 (106 nm) and 270 s^-1^mM^-1 ^(166 nm), respectively.

To demonstrate the presence of PAA, FT-IR of the clusters were performed (Figure 2A). Due to its coordination with iron during formation of the PAA-MNCs, the characteristic absorption of carboxylic group was not observed in the spectra [21]. The absorptions at 1600 and 1390 cm^-1 ^were assigned to the vibrations of carboxylate groups. The peak at 3500 cm^-1 ^was attributed to the absorbed water and that around 600 cm^-1 ^was vibration of Fe-O bond. To determine the mass fraction of magnetite in the clusters, thermogravimetric analysis (TGA) of clusters was recorded in Figure 2B. The weight loss below 200°C was attributed to the evaporation of the adsorbed water. From 200 to 900°C, the weight loss was ascribed to the decomposition of PAA chelated on the clusters. As the cluster size increased, the specific surface area decreased and the chelated PAA was reduced. As a result, with increase of cluster size, the mass fractions of iron oxide increased, which were 71, 81, 84, 91 wt% for 34, 63, 106, 166 nm clusters, respectively. The hydrodynamic size distributions of the clusters were characterized by dynamic laser scattering technology. The volume-weighted hydrodynamic sizes were 53, 95, 160, 236 nm for 34, 63, 106, 166 nm clusters, respectively (Figure 2C). The zeta potentials of the clusters at pH 7.0 were -38 mV (34 nm MNC), -43 mV (63 nm MNC), -47 mV (106 nm MNC) and -44 mV (166 nm MNC), respectively, which indicated that MNCs were highly negatively charged.

To determine the transverse relaxivity (r_2_) of clusters, the transverse relaxation times (T_2_) of the cluster water suspensions were measured by nuclear magnetic resonance (NMR). The measurement and calculation method of r_2 _were described in detail elsewhere [18]. Figure 2D displays the 1/T_2_, relaxation rate (R_2_), of the cluster suspensions *vs *different iron concentrations at logarithmic coordinates. The relaxation rates of 34, 63, 106, 166 nm MNCs at the iron concentration of 0.1 mM are 48, 62, 42, 27 s^-1^, respectively. The relaxivity (r_2_) of the clusters deduced from linear fitting of relaxation rates and iron concentrations were 490, 630, 420, 270 s^-1^mM^-1^, respectively, for 34 nm, 63 nm, 106 nm, 166 nm MNCs, much higher than those of the commercial contrast agents (usually less than 200 s^-1^mM^-1^) [22].

### In vitro study

**Figure 3:**
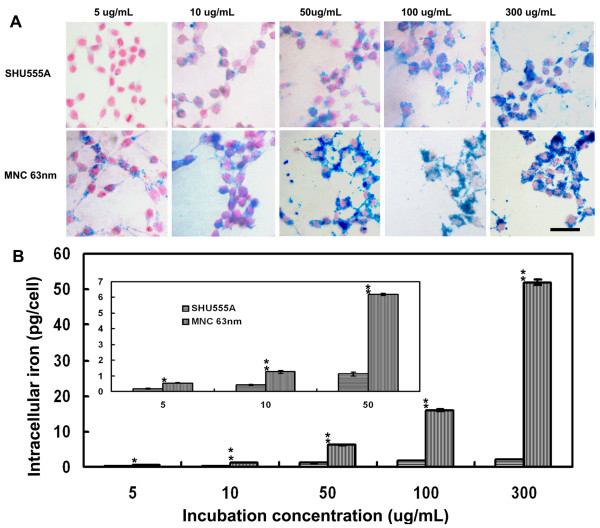
**Prussian blue staining (A) and ICP-OES quantification of intracellular iron contents (B) of RAW267.4 cell incubated with SHU555A or MNCs with average size of 63 nm at different iron concentrations for 1 h**. Cell uptake of both iron oxide particles are dose-dependent. However, cell incorporation of the MNCs is significantly higher than that of SHU555A. Bar: 20 μm. Inset (Figure B): Amplification of cell uptake at lower iron concentrations.

**Figure 4:**
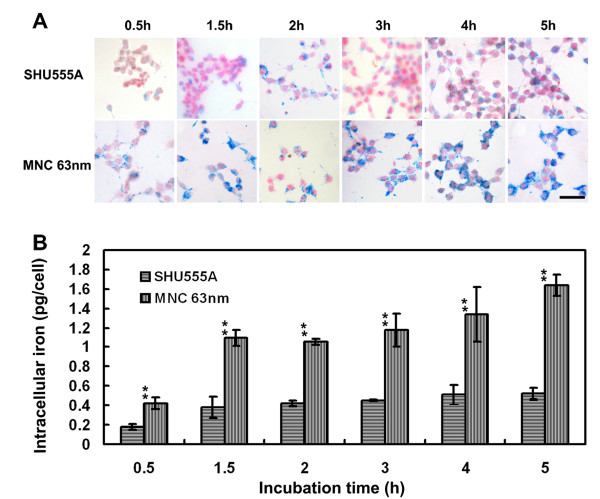
**Prussian blue staining (A) and ICP-OES quantification of intracellular iron contents (B) of RAW267.4 cell incubated with SHU555A or MNCs with average size of 63 nm at the concentration of 10 μg/mL for different periods of time**. Cell uptake of both iron oxide particles are time-dependent. However, cell incorporation of the MNCs are significantly higher than that of SHU555A. Bar: 20 μm.

Due to the high sensitivity of 63 nm MNCs, next we examine their potentials for MR cell imaging, with SHU555A (Resovist) as a reference. SHU555A is a clinically approved carboxydextran-coated SPION that is used as a negative MRI contrast agent for hepatic imaging [23] and also frequently used for cell imaging because of its high MR sensitivity [22,24]. As shown in Figure 3A and 4A, Prussian blue staining indicated cells incorporated both iron oxide particles in concentration- and time-dependent manners and the uptake rose with increase of concentrations or incubation times. The particles were unevenly localized in the periphery of nucleus.

For cell concentration-dependent uptake, a significant increase in intracellular iron was seen for MNCs already at an incubation concentration of 5 μg/mL (0.54 pg/cell) (Figure 3B). The intracellular iron contents more than doubled at the concentration of 10 μg/mL (1.25 pg/cell) and showed a further increase with further increase of concentrations. When the incubation concentration was at 300 μg/mL, the intracellular iron reached 52 pg/cell and it is seem that the uptake was still unsaturated. A similar concentration-dependent uptake was also observed for SHU555A, however, which was significant less than that of MNCs, and with increase of incubation concentrations, the difference between uptakes of the two kinds of iron oxide particles became more and more pronounced (Figure 3B).

For time-dependent uptake, incubation with the MNCs (10 μg/mL) led to a significant increase in intracellular iron already after 30 minutes (0.42 pg/cell). In comparison, the cell uptake of SHU555A was 0.18 pg/cell, less than half of that of MNCs. With increase of incubation times, cell incorporation of both iron oxide particles increased gradually (Figure 4B). However, cell incorporation of MNCs was more significant than that of SHU555A during each incubation time periods (p < 0.01). SHU555A is SPION with an Fe^2+ ^and Fe^3+ ^iron oxide core (4.2 nm) and a carboxydextran coating. SHU555A has a mean hydrodynamic diameter of 62 nm [25] and has been demonstrated highly efficient for cell labeling [26]. MNCs compose of individual SPION, which compact together densely. Moreover, the differences in coating materials and consequently different surface charges of SHU555A and MNC may also partially explain the different internalization efficiencies reported here. Therefore, the high efficiency of MNCs for cell uptake may arise from the different characteristics of the two kinds of iron oxide particles, and compact structure with high iron content and surface charge favors cell uptake.

**Figure 5:**
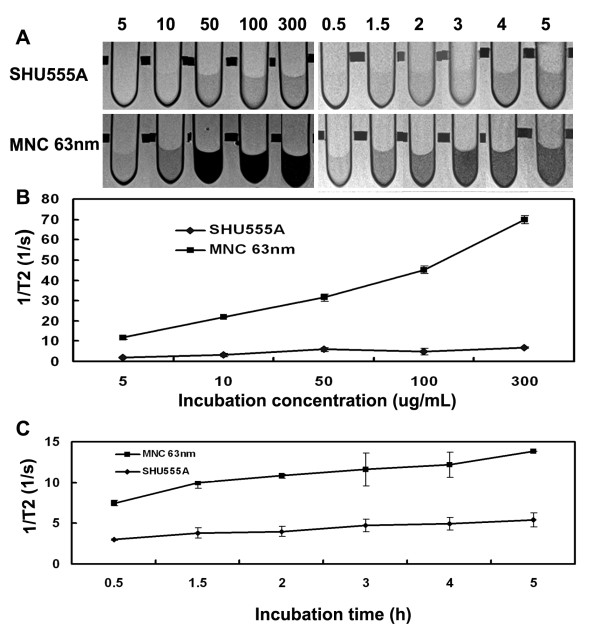
**T_2_-weighted MR imaging (A) and the corresponding T_2 _relaxation rates (1/T_2_) (B, C) of RAW267.4 cell incubated with SHU555A or the MNCs with average size of 63 nm at different concentrations for 1 h or at the concentration of 10 μg/mL for different periods of time**. For relaxation time measurements, 1 × 10^5 ^labeled cells were suspended in gelatin (2%, 500 μL) in plastic vials homogenously.

**Figure 6:**
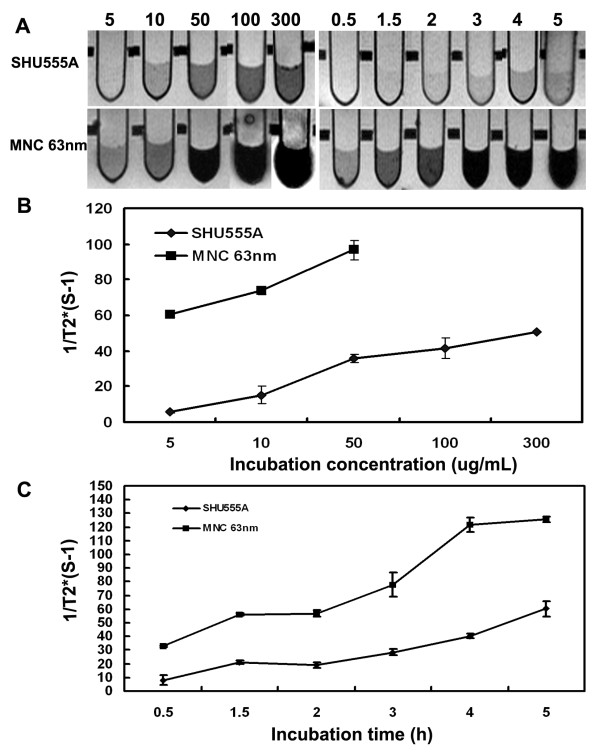
T_2_*-weighted MR imaging (A) and the corresponding T_2_* relaxation rates (1/T_2_*) (B, C) of RAW267.4 cell incubated with SHU555A or the MNCs with average size of 63 nm at different concentrations for 1 h or at the concentration of 10 μg/mL for different periods of time. For relaxation time measurements, 1 × 10^5 ^labeled cells were suspended in gelatin (2%, 500 μL) in plastic vials homogenously.

The efficacy of MNC for MR cell imaging was clearly demonstrated by T_2_- and T_2_*-weighted images of the labeled cells (Figure 5, 6). For cells treated with the MNCs at different concentrations for 1 h, the minimum concentration for cell labeling that made the labeled cells MR signal decrease detectable in both T_2_- and T_2_*-weighted images was 5 μg/mL (0.54 pg/cell). However, for cells treated with SHU555A, that was 50 μg/mL (1.11 pg/cell) for T_2_-weighted images and 10 μg/mL (0.42 pg/cell) for T_2_*-weighted images (Figure 5A, 6A). For cells treated with the MNCs at 10 μg/mL for different incubation time periods, the incubation times for MNCs for T_2_- and T_2_*-weighted imaging with significant MR signal decrease were also much shorter than those of SHU555A (Figure 5A, 6A). The corresponding T_2 _and T_2_* relaxation rates, R2 and R2*, were summarized in Figure 5B, C and 6B, C.

In current experiment setting, the detection limit of the MNCs for T_2_-wieghted imaging is 0.42 pg/cell (10 μg/mL, 0.5 h) and that for SHU555A is 0.51 pg/cell (10 μg/mL, 4 h) (Figure 5A). While, for T_2_*-weighted imaging, the detection limit is pretty similar, 0.42 pg/cell for the MNCs (10 μg/mL, 0.5 h) and 0.43 pg/cell for SHU555A (10 μg/mL, 3 h) (Figure 6A). These findings are consistent with previous reports that the detection sensitivity of T_2_-weighted imaging is governed by both sensitivity of magnetic particles and intracellular iron content, while for T_2_*-weighted imaging, it is mainly determined by intracellular iron content [27].

**Figure 7:**
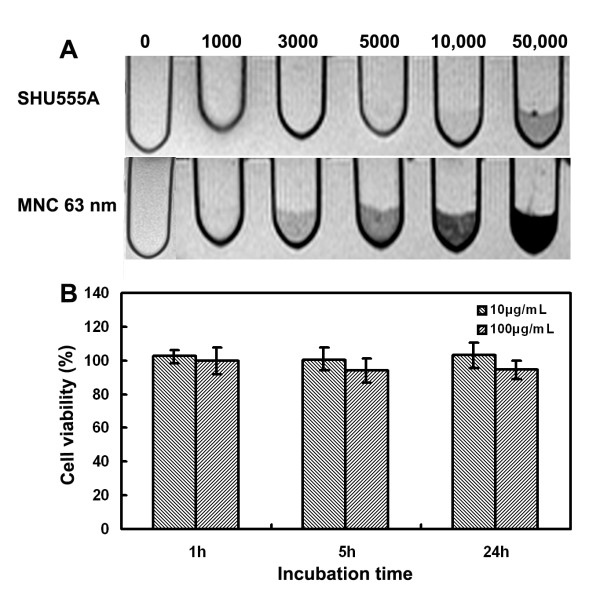
**A: T_2_-weighted MR images of different amounts of RAW267.4 cell treated with SHU555A or the MNCs with average size of 63 nm at the concentration of 100 μg/mL for 1 h**. The cells were suspended in 300 μL of 2% gelatin in plastic vials homogenously. B: Viability of RAW267.4 cell treated with the MNCs (60 nm) at the concentrations of 10 or 100 μg/mL for different periods of time.

Given the high efficiency of the MNCs for cell imaging, next we examined the detection threshold of MNC-labeled cells. For this purpose, the cells were treated with the MNCs at the concentration of 100 μg/mL for 1 h and were dispersed into 300 μL gelatin with different cell concentrations. For both the MNCs and SHU555A, the reduction in signal intensity depends on the cell number. For MNC-labeled cells, the hypointense signal was discernable at 3,000 cells. As a contrast, for SHU555A-treated cells, more cells are necessary (50,000 cells) to produce a visible signal reduction (Figure 7A). These observations further indicated that the MNCs are much more efficient for MR cell imaging than SHU555A.

As regards the cell viability, no toxic effects were noted for the MNCs at lower iron concentration (10 μg/mL), reaching 102.56 ± 4.04% (1 h), 100.60 ± 6.75% (5 h) and 103.07 ± 7.31% (24 h) viability. At higher iron concentration (100 μg/mL), cell viability decreased slightly with increase of incubation times, which were 99.86 ± 4.04%, 93.97 ± 6.94% and 94.56 ± 5.20% for incubation 1 h, 5 h and 24 h, respectively (Figure 7B).

### In vivo study

**Figure 8:**
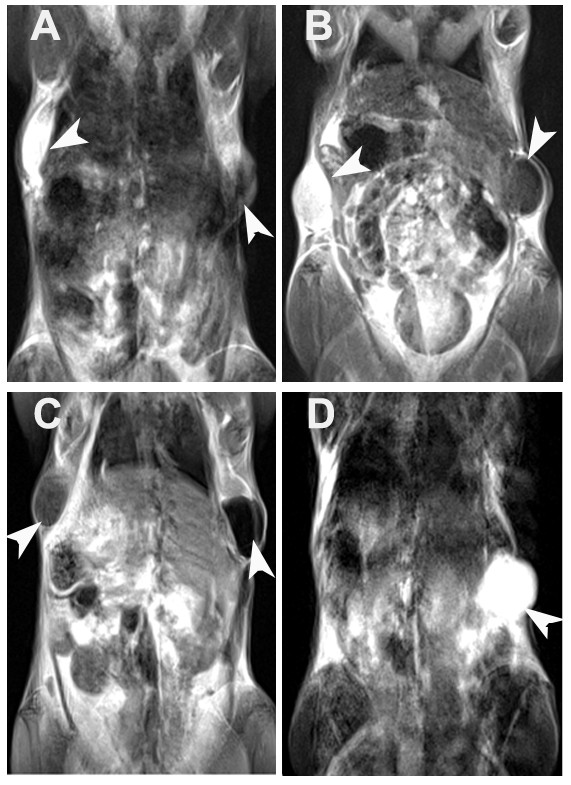
**T_2_-weighted MR images of different amounts of RAW267.4 cell (treated with 100 μg/mL particles for 1 h) suspended in Matrigel (0.4 mL) and embedded subcutaneously into the dorsal flanks of mice (arrow head)**. A: 1 × 10^4^. B: 1 × 10^5^. C: 1 × 10^6 ^. Left side: cells treated with SHU555A. Right side: cells treated with the MNCs (63 nm). D: plain Matrigel.

Finally, we conducted MR imaging of MNC-treated cells in a mouse model. Different amounts of cells (1 × 10^4^, 1 × 10^5^, 1 × 10^6^) treated with 100 μg/mL of the MNCs or SHU555A for 1 h were suspended in Matrigel (0.4 mL) and injected subcutaneously into the dorsal flanks of each mouse to mimic the in vivo condition (Figure 8). For T_2_-weighted images, cells treated with the MNCs could be detected at the dorsal flanks of the mice at the cell number of 1 × 10^4 ^and presented as a dark, protruding mass, while the same amount of cells treated with SHU555A could only be detected as a bright dot, a signal which only reflects Matrigel (Figure 8A). With increase of cells implanted, the dark signal became more and more intense, and cells treated with SHU555A could only be detected at cell number of 1 × 10^6 ^(Figure 8C). The findings revealed that the MNCs were also efficient for cell imaging under in vivo condition and the labeled cells could be detected in a dose-responsive manner in living animals under a clinical MRI system.

## Conclusions

In current study, we have prepared compact magnetic nanoclusters (MNCs) with different sizes by polyol method and the MNCs with average size of 63 nm demonstrate the highest MR sensitivity (630 s^-1^mM^-1^). When used for cell imaging with SHU555A as a reference, both iron oxide particles were incorporated by RAW264.7 cells in a concentration- and time-dependent manner. However, cell uptake of the MNCs is more efficient than that of SHU555A. MR imaging indicated that the MNCs demonstrated superior sensitivity than SHU555A both in vitro and in vivo, with detection limits of 3,000 and 10,000 labeled cells, respectively. Our study indicated that the MNCs are sensitive for cell imaging and promising for MR cell tracking in future.

## Competing interests

The authors declare that they have no competing interests.

## Authors' contributions

ML carried out all the experiments and drafted the manuscript. HG and CZ contributed to conception of the study and to interpretation of data, and revised the manuscript. Both authors read and approved the final manuscript.
